# Supplementary material for: Overlapping cell population expression profiling and regulatory inference in C. elegans
Source: BMC Genomics. 2016 Feb 29;17:159. doi: 10.1186/s12864-016-2482-z (PMC4772325; doi:10.1186/s12864-016-2482-z)
Supplement: Additional file 13: — Web supplement. (DOC 21 kb) [file 12864_2016_2482_MOESM13_ESM.zip › sortWeb/clusters/hier.300.clusters/97.html]

Cluster 97 

## Cluster 97

### Expression

| cnd-1 rep. 1 | cnd-1 rep. 2 | cnd-1 rep. 3 | pha-4 rep. 1 | pha-4 rep. 2 | pha-4 rep. 3 | ceh-27 | ceh-36 | ceh-6 | F21D5.9 | mir-57 | mls-2 | pal-1 | pros-1 | ttx-3 | unc-130 | hlh-16 | irx-1 | ceh-6 (+) hlh-16 (+) | ceh-6 (+) hlh-16 (-) | ceh-6 (-) hlh-16 (+) | cnd-1 singlets | pha-4 singlets | 0 | 60 | 120 | 150 | 180 | 240 | 330 | 390 | 420 | 480 | 540 | 570 | 600 | 630 | 660 | NAME | Functional description |
| --- | --- | --- | --- | --- | --- | --- | --- | --- | --- | --- | --- | --- | --- | --- | --- | --- | --- | --- | --- | --- | --- | --- | --- | --- | --- | --- | --- | --- | --- | --- | --- | --- | --- | --- | --- | --- | --- | --- | --- |
|  |  |  |  |  |  |  |  |  |  |  |  |  |  |  |  |  |  |  |  |  |  |  |  |  |  |  |  |  |  |  |  |  |  |  |  |  |  | M60.12 |  |
|  |  |  |  |  |  |  |  |  |  |  |  |  |  |  |  |  |  |  |  |  |  |  |  |  |  |  |  |  |  |  |  |  |  |  |  |  |  | C14B9.12 |  |
|  |  |  |  |  |  |  |  |  |  |  |  |  |  |  |  |  |  |  |  |  |  |  |  |  |  |  |  |  |  |  |  |  |  |  |  |  |  | Y113G7A.19 |  |
|  |  |  |  |  |  |  |  |  |  |  |  |  |  |  |  |  |  |  |  |  |  |  |  |  |  |  |  |  |  |  |  |  |  |  |  |  |  | C17H12.32 |  |
|  |  |  |  |  |  |  |  |  |  |  |  |  |  |  |  |  |  |  |  |  |  |  |  |  |  |  |  |  |  |  |  |  |  |  |  |  |  | C14A11.10 |  |
|  |  |  |  |  |  |  |  |  |  |  |  |  |  |  |  |  |  |  |  |  |  |  |  |  |  |  |  |  |  |  |  |  |  |  |  |  |  | T22B2.10 |  |
|  |  |  |  |  |  |  |  |  |  |  |  |  |  |  |  |  |  |  |  |  |  |  |  |  |  |  |  |  |  |  |  |  |  |  |  |  |  | Y53G8B.t1 |  |
|  |  |  |  |  |  |  |  |  |  |  |  |  |  |  |  |  |  |  |  |  |  |  |  |  |  |  |  |  |  |  |  |  |  |  |  |  |  | C17H12.34 |  |
|  |  |  |  |  |  |  |  |  |  |  |  |  |  |  |  |  |  |  |  |  |  |  |  |  |  |  |  |  |  |  |  |  |  |  |  |  |  | *srx-61* | Serpentine Receptor, class X |
|  |  |  |  |  |  |  |  |  |  |  |  |  |  |  |  |  |  |  |  |  |  |  |  |  |  |  |  |  |  |  |  |  |  |  |  |  |  | C14C10.9 |  |
|  |  |  |  |  |  |  |  |  |  |  |  |  |  |  |  |  |  |  |  |  |  |  |  |  |  |  |  |  |  |  |  |  |  |  |  |  |  | Y25C1A.14 |  |
|  |  |  |  |  |  |  |  |  |  |  |  |  |  |  |  |  |  |  |  |  |  |  |  |  |  |  |  |  |  |  |  |  |  |  |  |  |  | *srbc-3* | Serpentine Receptor, class BC (class B-like) |
|  |  |  |  |  |  |  |  |  |  |  |  |  |  |  |  |  |  |  |  |  |  |  |  |  |  |  |  |  |  |  |  |  |  |  |  |  |  | *col-20* | COLlagen |
|  |  |  |  |  |  |  |  |  |  |  |  |  |  |  |  |  |  |  |  |  |  |  |  |  |  |  |  |  |  |  |  |  |  |  |  |  |  | *col-171* | COLlagen |
|  |  |  |  |  |  |  |  |  |  |  |  |  |  |  |  |  |  |  |  |  |  |  |  |  |  |  |  |  |  |  |  |  |  |  |  |  |  | *srh-17* | Serpentine Receptor, class H |
|  |  |  |  |  |  |  |  |  |  |  |  |  |  |  |  |  |  |  |  |  |  |  |  |  |  |  |  |  |  |  |  |  |  |  |  |  |  | F14B8.11 |  |
|  |  |  |  |  |  |  |  |  |  |  |  |  |  |  |  |  |  |  |  |  |  |  |  |  |  |  |  |  |  |  |  |  |  |  |  |  |  | Y87G2A.20 |  |
|  |  |  |  |  |  |  |  |  |  |  |  |  |  |  |  |  |  |  |  |  |  |  |  |  |  |  |  |  |  |  |  |  |  |  |  |  |  | K10D6.23 |  |
|  |  |  |  |  |  |  |  |  |  |  |  |  |  |  |  |  |  |  |  |  |  |  |  |  |  |  |  |  |  |  |  |  |  |  |  |  |  | ZC513.14 |  |
|  |  |  |  |  |  |  |  |  |  |  |  |  |  |  |  |  |  |  |  |  |  |  |  |  |  |  |  |  |  |  |  |  |  |  |  |  |  | F43G6.14 |  |
|  |  |  |  |  |  |  |  |  |  |  |  |  |  |  |  |  |  |  |  |  |  |  |  |  |  |  |  |  |  |  |  |  |  |  |  |  |  | H06A10.1 |  |
|  |  |  |  |  |  |  |  |  |  |  |  |  |  |  |  |  |  |  |  |  |  |  |  |  |  |  |  |  |  |  |  |  |  |  |  |  |  | M163.7 |  |
|  |  |  |  |  |  |  |  |  |  |  |  |  |  |  |  |  |  |  |  |  |  |  |  |  |  |  |  |  |  |  |  |  |  |  |  |  |  | F53B1.t1 |  |

### Phenotypes enriched

none found

### Anatomy terms enriched

none found

### GO terms enriched

none found

### Expression clusters enriched

none found

### Motifs enriched

|  |  |  |  |  |  |
| --- | --- | --- | --- | --- | --- |
| **Motif** | **Logo** | **Possible orthologs** | **Number of motifs in cluster** | **Enrichment** | **FDR corrected p** |
| V$RFX1\_01 |  | daf-19 | 10 | 7.98 | 4.7e-05 |
| MA0531.1 |  | Y5F2A.4 | 10 | 6.73 | 1.8e-04 |
| MA0007.2 |  | nhr-255 | 11 | 5.03 | 5.6e-04 |
| pTH9911 |  | atf-5 fos-1 | 13 | 3.79 | 9.0e-04 |
| pTH9708 |  | ceh-34 | 5 | 14.16 | 3.2e-03 |
| inv\_SOLEXA\_2\_FBgn0001269 |  | ceh-16 alr-1 ZC123.3 | 13 | 3.20 | 4.2e-03 |
| pTH9199 |  | mab-3 daf-19 | 14 | 2.92 | 4.6e-03 |
| HXC8\_f1 |  | lin-39 | 14 | 2.80 | 6.7e-03 |
| pTH6556 |  | lim-6 | 8 | 5.40 | 7.0e-03 |
| pTH6524 |  | ceh-32 | 12 | 3.18 | 9.3e-03 |
| NR4A1\_f1 |  | nhr-6 | 8 | 5.08 | 9.9e-03 |
| IRX5\_1 |  | irx-1 | 12 | 3.12 | 1.1e-02 |
| Six2\_2307 |  | ceh-32 | 12 | 3.09 | 1.2e-02 |
| HNF4G\_f1 |  | nhr-62 | 14 | 2.61 | 1.2e-02 |
| PBDE\_GATA1\_UCD |  | elt-1 | 14 | 2.60 | 1.3e-02 |
| Mw164 |  | lin-31 | 6 | 7.11 | 1.4e-02 |
| pTH9916 |  | W08E12.1 | 11 | 3.27 | 1.5e-02 |
| pTH10772 |  | ceh-52 | 5 | 9.20 | 1.6e-02 |
| MEIS1\_1 |  | ceh-32 | 9 | 3.91 | 1.9e-02 |
| foxo\_SANGER\_10\_FBgn0038197 |  | daf-16 | 7 | 5.27 | 2.0e-02 |
| MA0102.3 |  | C48E7.11 | 10 | 3.42 | 2.2e-02 |
| Jundm2\_0911 |  | fos-1 | 13 | 2.58 | 2.6e-02 |
| pTH9915 |  | zip-3 | 13 | 2.56 | 2.6e-02 |
| pTH9924 |  | nhr-46 | 5 | 7.82 | 2.8e-02 |
| YBOX1\_f2 |  | dro-1 cey-3 | 10 | 3.27 | 2.9e-02 |
| pTH10656 |  | hlh-12 | 10 | 3.26 | 2.9e-02 |
| SOX2\_2 |  | sox-4 | 5 | 7.63 | 3.0e-02 |
| pTH9322 |  | nhr-10 nhr-7 | 7 | 4.78 | 3.1e-02 |
| MSX1\_2 |  | ceh-1 | 13 | 2.51 | 3.2e-02 |
| HepG2\_HSF1\_Stanford |  | Y53C10A.3 | 8 | 4.03 | 3.3e-02 |
| K562b\_GATA2\_UCD |  | elt-1 | 5 | 7.29 | 3.5e-02 |
| pTH9323 |  | odr-7 | 10 | 3.13 | 3.7e-02 |
| pTH8982 |  | ceh-48 | 5 | 7.16 | 3.7e-02 |
| EGR1\_1 |  | ZC328.2 | 8 | 3.91 | 3.8e-02 |
| Scr\_Cell\_FBgn0003339 |  | lin-39 | 9 | 3.38 | 4.3e-02 |
| RFX3\_f1 |  | daf-19 | 10 | 3.01 | 4.6e-02 |

### Correlated (and anti-correlated) transcription factors

|  |  |
| --- | --- |
| **Transcription factor** | **Correlation** |
| rnt-1 | 0.69 |
| ehn-3 | 0.65 |
| K05F1.5 | 0.65 |
| lir-1 | 0.64 |
| ceh-99 | 0.64 |
| lin-26 | 0.59 |
| nhr-25 | 0.58 |
| egl-5 | 0.58 |
| T22C8.4 | 0.57 |
| hlh-8 | 0.52 |
| nhr-69 | 0.52 |
| hlh-1 | 0.51 |
| dmd-3 | 0.51 |
| nhr-111 | 0.51 |
| tlp-1 | 0.51 |
| mls-1 | 0.51 |
| unc-120 | 0.50 |
| unc-98 | 0.50 |
| nhr-9 | 0.50 |
| pat-9 | 0.49 |
| pal-1 | 0.49 |
| mab-5 | 0.49 |
| blmp-1 | 0.49 |
| nob-1 | 0.49 |
| dsc-1 | 0.48 |
| nhr-207 | -0.35 |
| nhr-134 | -0.36 |
| sem-2 | -0.36 |
| mnm-2 | -0.36 |
| dhhc-12 | -0.36 |
| dmd-5 | -0.36 |
| nhr-84 | -0.36 |
| nhr-71 | -0.37 |
| egrh-2 | -0.37 |
| pzf-1 | -0.37 |
| nhr-54 | -0.38 |
| nhr-275 | -0.38 |
| ceh-34 | -0.39 |
| ceh-36 | -0.39 |
| mef-2 | -0.39 |
| nhr-285 | -0.40 |
| F17C11.1 | -0.40 |
| K11D12.12 | -0.41 |
| nhr-213 | -0.41 |
| nhr-215 | -0.43 |
| R05D3.3 | -0.44 |
| nhr-65 | -0.45 |
| nhr-116 | -0.46 |
| nhr-192 | -0.48 |
| fkh-2 | -0.52 |

### ChIP peaks enriched

none found
